# Molecular analysis of sunitinib resistant renal cell carcinoma cells after sequential treatment with RAD001 (everolimus) or sorafenib

**DOI:** 10.1111/jcmm.12471

**Published:** 2014-12-02

**Authors:** Eva Juengel, Dana Kim, Jasmina Makarević, Michael Reiter, Igor Tsaur, Georg Bartsch, Axel Haferkamp, Roman A Blaheta

**Affiliations:** Department of Urology, Johann Wolfgang Goethe-UniversityFrankfurt am Main, Germany

**Keywords:** Sequential therapy, renal cell carcinoma, RAD001, sunitinib, sorafenib

## Abstract

Sequential application of target drugs is standard procedure after renal cell carcinoma (RCC) patients develop resistance. To optimize the sequence, antitumour effects of the mTOR inhibitor RAD001 or the tyrosine kinase inhibitor (TKI) sorafenib on RCC cells with acquired resistance to the TKI sunitinib was evaluated. RCC cells were exposed to 1 μM sunitinib for 24 hrs (as control) and for 8 weeks (to induce resistance) and then switched to RAD001 (5 nM) or sorafenib (5 μM) for a further 8 weeks. Tumour cell growth, cell cycle progression, cell cycle regulating proteins and intracellular signalling were then investigated. Short-term application of sunitinib (24 hrs) induced cell growth blockade with accumulation in the G2/M phase. RCC cells became resistant to sunitinib after 8 weeks, demonstrated by accelerated cell growth along with enhanced cdk1, cdk2, loss of p27, activation of Akt, Rictor and Raptor. Switching to sorafenib only slightly reduced growth of the sunitinib resistant RCC cells and molecular analysis indicated distinct cross-resistance. In contrast, full response was achieved when the cancer cells were treated with RAD001. p19 and p27 strongly increased, phosphorylated Akt, Rictor and Raptor decreased and the tumour cells accumulated in G0/G1. It is concluded that an mTOR-inhibitor for second-line therapy could be the strategy of choice after first-line sunitinib failure.

## Introduction

The development of targeted drugs has led to significant improvement in the prognosis of metastatic renal cell carcinoma (RCC). The multi-targeted tyrosine kinase inhibitor (TKI) sunitinib, which exerts its antitumour effects primarily through the selective inhibition of VEGF receptor (VEGFR) has been approved by the United States Food and Drug Administration and by the European Medicines Agency as first-line treatment for RCC-patients with good or intermediate prognosis 1. A further TKI, sorafenib, has been authorized for treating patients with advanced RCC, for whom prior interferon-alpha or interleukin-2 based therapy failed or who were considered unsuitable for such therapy 1. The mammalian target of rapamycin (mTOR) inhibitor temsirolimus has been approved for the first-line treatment of RCC patients with poor-prognosis, whereas the oral mTOR-inhibitor RAD001 (everolimus) has been recommended for patients with advanced progressive RCC or for patients with failed VEGF-targeted therapy 2.

Unfortunately, the strategy of tumour targeting is rarely curative. It has been argued that the tumour may adapt to chronic drug use and avoid drug mediated growth control. To overcome this obstacle, sequential therapy is considered an innovative option providing maximal efficacy with a minimum risk of therapeutic failure. Still, it remains unclear which compound is best applied after patients have become resistant to a TKI based regimen. Hypothetically, this could be an alternative TKI, which may act on similar pathways as the first-line TKI, or an mTOR-inhibitor, which might alter intracellular signalling pathways different from the one targeted by the first-line TKI 3.

Prospective trials comparing a TKI-TKI with a TKI-mTOR-inhibitor sequence have not yet been published 4,5. We, therefore, have initiated a preclinical study to compare the antitumour potential of sorafenib *versus* RAD001 in a second line setting. RCC cells, which have been driven to sunitinib-resistance were treated with sorafenib or RAD001 for different time periods and the biological as well as the molecular responses were investigated. Our data point to distinct differences between the sorafenib and the RAD001 based regimen. Sorafenib only slightly counteracted resistance effects caused by sunitinib and only moderately diminished RCC tumour growth, compared to its influence on sunitinib-sensitive cells. In contrast, RAD001 evoked a strong response of the sunitinib-resistant RCC cells, which was similar to the one seen in sunitinib-sensitive cells. Molecular analysis revealed cross-resistance between sunitinib and sorafenib, which might be responsible for the limited effect observed with second line sorafenib treatment.

## Materials and methods

### Cell culture

Kidney carcinoma Caki-1 and KTC-26 cells were purchased from LGC Promochem (Wesel, Germany). A498 cells were derived from Cell Lines Service (Heidelberg, Germany). Tumour cells were grown and subcultured in RPMI 1640 medium (Seromed, Berlin, Germany) supplemented with 10% FCS, 100 IU/ml penicillin and 100 μg/ml streptomycin (all Gibco/Invitrogen, Karlsruhe, Germany) at 37°C in a humidified, 5% CO_2_ incubator.

### Drugs

RAD001 (provided by Novartis Pharma AG, Basel, Switzerland) was dissolved in DMSO (Merck, Darmstadt, Germany) as 10 mM stock solution and stored in aliquots at −20°C. Prior to the experiments, RAD001 was diluted in cell culture medium to a final concentration of 5 nM. Sunitinib and sorafenib were from LC Laboratories, Woburn, MA, USA, and used at a final concentration of 1 μM (sunitinib) or 5 μM (sorafenib).

Renal cell carcinoma cell lines were treated twice a week with sunitinib over a period of 8 weeks. Subsequently, sunitinib was replaced by sorafenib or RAD001 for a further period of 8 weeks. Both sorafenib and RAD001 were applied twice a week. Control cells received cell culture medium alone or sunitinib for a total of 16 weeks. Additionally, fresh cells, not pre-treated with sunitinib, were exposed to sorafenib or RAD001 to investigate the maximum effect of RAD001 and sorafenib. The strategy of chronic drug treatment with a constant, instead of an increasing dosage was based on an earlier study, whereby this protocol proved to initiate resistance 6.

Cell viability was determined by trypan blue (Gibco/Invitrogen, Karlsruhe, Germany) 1 day and 8 weeks after sunitinib application, and 1 day and 8 weeks after sorafenib or RAD001 application. Cell viability was also controlled at every cell passage. For all further tests, tumour cells were subjected to the assays listed below 1 day and 8 weeks after sunitinib application, and 1 day and 8 weeks after sorafenib or RAD001 application.

### Apoptosis

To detect apoptosis the expression of Annexin V/propidium iodide (PI) was evaluated using the Annexin V-FITC Apoptosis Detection kit (BD Pharmingen, Heidelberg, Germany). Tumour cells were washed twice with PBS, and then incubated with 5 μl of Annexin V-FITC and 5 μl of PI in the dark for 15 min. at RT. Cells were analysed on a FACScalibur (BD Biosciences, Heidelberg, Germany). The percentage of apoptotic cells (early and late) in each quadrant was calculated using CellQuest software (BD Biosciences). Caspase-3, Bcl-2 and Bax expression were additionally evaluated by Western blotting using the following antibodies: Anti-caspase-3 (#9662; Cell Signalling-Millipore, Darmstadt, Germany), Anti-Bcl-2 (clone N-19), Anti-Bax (clone N-20, both Santa Cruz, Heidelberg, Germany).

### Measurement of tumour cell growth

Cell growth was assessed using the 3-(4,5-dimethylthiazol-2-yl)-2,5-diphenyltetrazolium bromide (MTT) dye reduction assay (Roche Diagnostics, Penzberg, Germany). Caki-1 cells (50 μl, 1 × 10^5^ cells/ml) were seeded onto 96-well tissue culture plates. After 24, 48 and 72 hrs, 10 μl MTT (0.5 mg/ml) were added for an additional 4 hrs. Thereafter, cells were lysed in a buffer containing 10% SDS in 0.01 M HCl. The plates were incubated overnight at 37°C, 5% CO_2_. Absorbance at 550 nm was determined for each well using a microplate ELISA reader. A standard curve was run in parallel to calculate the cell number, assuming that mitochondrial activity was the same in all the cell cultures. Each experiment was done in triplicate. After subtracting background absorbance, results were expressed as mean cell number.

### Cell cycle analysis

Cell cycle analysis was carried out on cell cultures grown to subconfluency. Tumour cell populations were stained with PI, using a Cycle TEST PLUS DNA Reagent Kit (BD Pharmingen) and then subjected to flow cytometry with a FACScan flow cytometer (BD Biosciences). 10,000 events were collected from each sample. Data acquisition was carried out using Cell-Quest software and cell cycle distribution was calculated using the ModFit software (BD Biosciences). The number of gated cells in G1, G2/M or S-phase was expressed as %.

### Western blot analysis of cell cycle regulating proteins

To explore cell cycle regulating proteins, tumour cell lysates were applied to a 7% polyacrylamide gel and electrophoresed for 90 min. at 100 V. The lysis buffer consisted of Tris-Nacl, 10% Tergitol, 0.25% Na-deoxycholate, 1 mM EDTA, 1 mg/ml aprotinin, 1 mg/ml leupeptin, 1 mg/ml pepstatin, 2 mM NaF, 2 mM Na_3_VO_4_, 2 mM PMSF. The protein was then transferred to nitrocellulose membranes (1 hr, 100 V). After blocking with non-fat dry milk for 1 hr, the membranes were incubated overnight with monoclonal antibodies directed against the following cell cycle proteins (all from BD Biosciences): Cdk1 (IgG1, clone 1), cdk2 (IgG2a, clone 55), cdk4 (IgG1, clone 97), cyclin A (IgG1, clone 25), cyclin B (IgG1, clone 18), cyclin D1 (IgG1, clone G124-326), cyclin E (IgG1, clone HE12), p19 (IgG1, clone 52/p19), p27 (IgG1, clone 57). HRP-conjugated goat-anti-mouse IgG (Upstate Biotechnology, Lake Placid, NY, USA; dilution 1:5000) served as the secondary antibody. The membranes were briefly incubated with ECL detection reagent (ECL™, Amersham/GE Healthcare, München, Germany) to visualize the proteins and then analysed by the Fusion FX7 system (Peqlab, Erlangen, Germany). β-actin (1:1000; Sigma-Aldrich, Taufenkirchen, Germany) served as the internal control.

Gimp 2.8 software was used to perform pixel density analysis of the protein bands. The ratio of protein intensity/β-actin intensity was calculated, and expressed in percentage, related to controls set to 100%.

### Expression and activity of cell signalling proteins

To explore the expression level of specific targets related to sunitinib, sorafenib and RAD001, Caki-1 cells (treated *versus* controls) were kept in serum-free cell culture medium overnight and then stimulated for 30 min. with EGF (Promocell GmbH, Heidelberg, Germany; 100 ng/ml) 7. Western blotting was carried out thereafter, using the following monoclonal antibodies from New England Biolabs GmbH, Frankfurt, Germany: Anti VEGFR2 (IgG, 55B11), Anti phospho VEGFR2 (IgG, D5A6), Anti Rictor (IgG, D16H9), Anti phospho Rictor (IgG, Thr1135, D30A3), Anti Raptor (IgG, 24C12), Anti phospho Raptor (IgG, Ser792). Anti EGFR (IgG1, clone 13/EGFR), Anti phospho EGFR (pEGFR; IgG1, clone 74), Anti Akt (IgG1, clone 55), Anti phospho Akt (pAkt; IgG1, clone 104A282) were from BD Pharmingen. Since the mTOR complex is composed of two subunits, Rictor (mTORC2) and Raptor (mTORC1), whereby Raptor is considered sensitive and Rictor insensitive to rapamycin, both units, instead of total mTOR, were analysed.

Gimp 2.8 software was used to perform pixel density analysis of the protein bands. The ratio of protein intensity/β-actin intensity was calculated, and expressed in percentage, related to controls set to 100%.

### Statistics

All experiments were performed 3–6 times. Statistical significance was determined with the Wilcoxon-Mann–Whitney *U*-test. Differences were considered statistically significant at a *P*-value less than 0.05.

## Results

### Drug dosage

**Fig 1:**
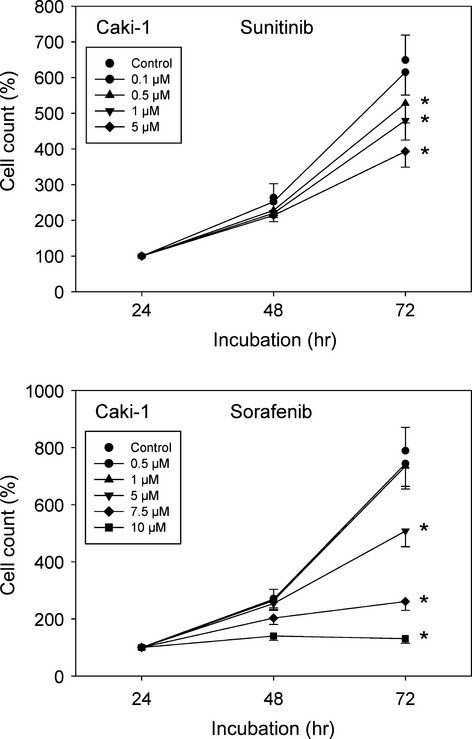
Dose-response analysis. Caki-1 cells were treated with various concentrations of sunitinib or sorafenib or remained un-treated (control). Cell numbers at 24 hrs were set to 100%. One representative of 6 experiments is shown. * indicates significant difference to controls.

Based on earlier studies, RAD001 was applied at a 5 nM concentration 6,8. To determine the optimum sunitinib and sorafenib dosage, all RCC cell lines were treated with different drug concentrations and cell growth was evaluated. 0.1 μM sunitinib did not exert a significant growth blocking effect, whereas 0.5, 1 and 5 μM did (Fig.1, representative for Caki-1). Signs of toxicity became apparent when tumour cells were exposed to 10 μM sunitinib (data not shown). All further experiments were, therefore, carried out with 1 μM sunitinib. Sorafenib caused a distinct down-regulation of the tumour cell number at a dosage of ≥5 μM (Fig.1, representative for Caki-1). Therefore, 5 μM sorafenib was used in the present investigation.

### Resistance induction by sunitinib

**Fig 2:**
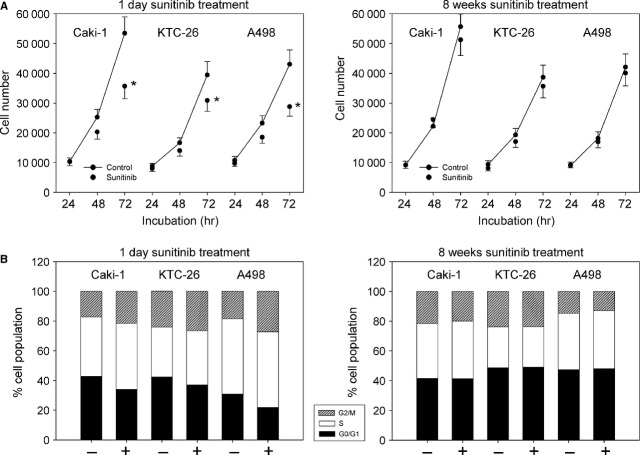
(A) Cell growth analysis of Caki-1, KTC-26 and A498 cells treated short-term (1 day) or long-term (8 weeks) with 1 μM sunitinib. Controls remained untreated. The figure shows one representative from six separate experiments. *indicates significant difference to controls. (B) Cell cycle analysis of Caki-1, KTC-26 and A498 cells treated short-term (1 day) or long-term (8 weeks) with 1 μM sunitinib. The cell population at each specific checkpoint is expressed as percentage of the total cells analysed. – indicates controls + indicates sunitinib treatment. One representative experiment of three is shown.

When Caki-1, KTC-26 or A498 cells were treated with 1 μM sunitinib for 1 day cell growth, assessed by the MTT-assay, was significantly reduced (Fig.2A, left). In parallel, the number of RCC cells in the G2/M-phase was significantly elevated, whereas the number of RCC cells in the G0/G1-phase was significantly diminished (Fig.2B, left).

Exposing the tumour cells to 1 μM sunitinib for 8 weeks resulted in drug non-responsiveness, reflected in tumour cell number being equal to untreated controls (Fig.2A, right). This contrasts with the early effects of sunitinib where a distinct growth inhibitory effect (Fig.2A, left) was evoked. The same was true with respect to cell cycle progression, which after 8 weeks no longer revealed differences between treated and untreated tumour cells (Fig.2B, right).

**Fig 3:**
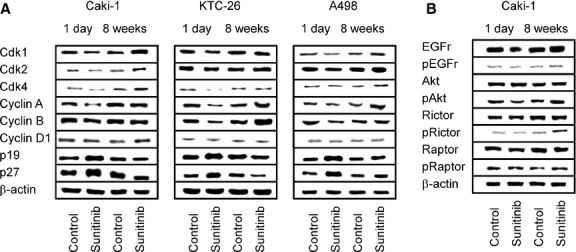
Western blot analysis of cell cycle related (A) or cell signalling (B) proteins, listed in methods. RCC cells were treated short-term (1 day) or long-term (8 weeks) with 1 μM sunitinib. Controls remained untreated. β-actin served as the internal control. The figure shows one representative from three separate experiments.

The cell cycle regulating proteins cdk1, cdk2 (not in KTC-26), cdk4 (not in A498), cyclin A and cyclin B were all down-regulated in the tumour cells when treated for 24 hrs with sunitinib (Fig.3A). Cyclin D1 was not influenced by sunitinib, and cyclin E was not detectable in Caki-1, KTC-26 or A498 cells. Short-term sunitinib treatment led to a considerable increase of p19 and p27 proteins. Chronic administration of sunitinib over 8 weeks reversed the processes triggered by sunitinib after 24 hrs. Caki-1, cdk1, cdk2 and cdk4 were then elevated, and p27 was diminished with sunitinib, compared to the controls. No difference to the controls was induced with respect to cyclin A, cyclin B and p19 (Fig.3A). KTC-26, cyclin A and cyclin B were elevated, p19 and p27 diminished, compared to the controls after 8 weeks. In A498 cells a similar expression pattern of the cdk-cyclin members and the proteins p19 and p27 was detected in treated and non-treated cells (Fig.3A).

Cell signalling was further explored in Caki-1 cells. Both total and phosphorylated VEGFR were not detected in this cell line (data not shown). EGFR was strongly expressed in the Caki-1 controls and became further elevated after 8 weeks chronic sunitinib exposure. The active form, pEGFR, was only marginally detected, with a slight increase in Caki-1 treated for 8 weeks with sunitinib (Fig.3B). Sunitinib induced a moderate reduction of pAkt after 24 hrs, but not of the mTOR components, pRictor and pRaptor. pAkt, pRictor and pRaptor increased in the tumour cells when driven to resistance (8 weeks analysis, Fig.3B).

### Sequential switch to sorafenib following sunitinib-resistance

**Fig 4:**
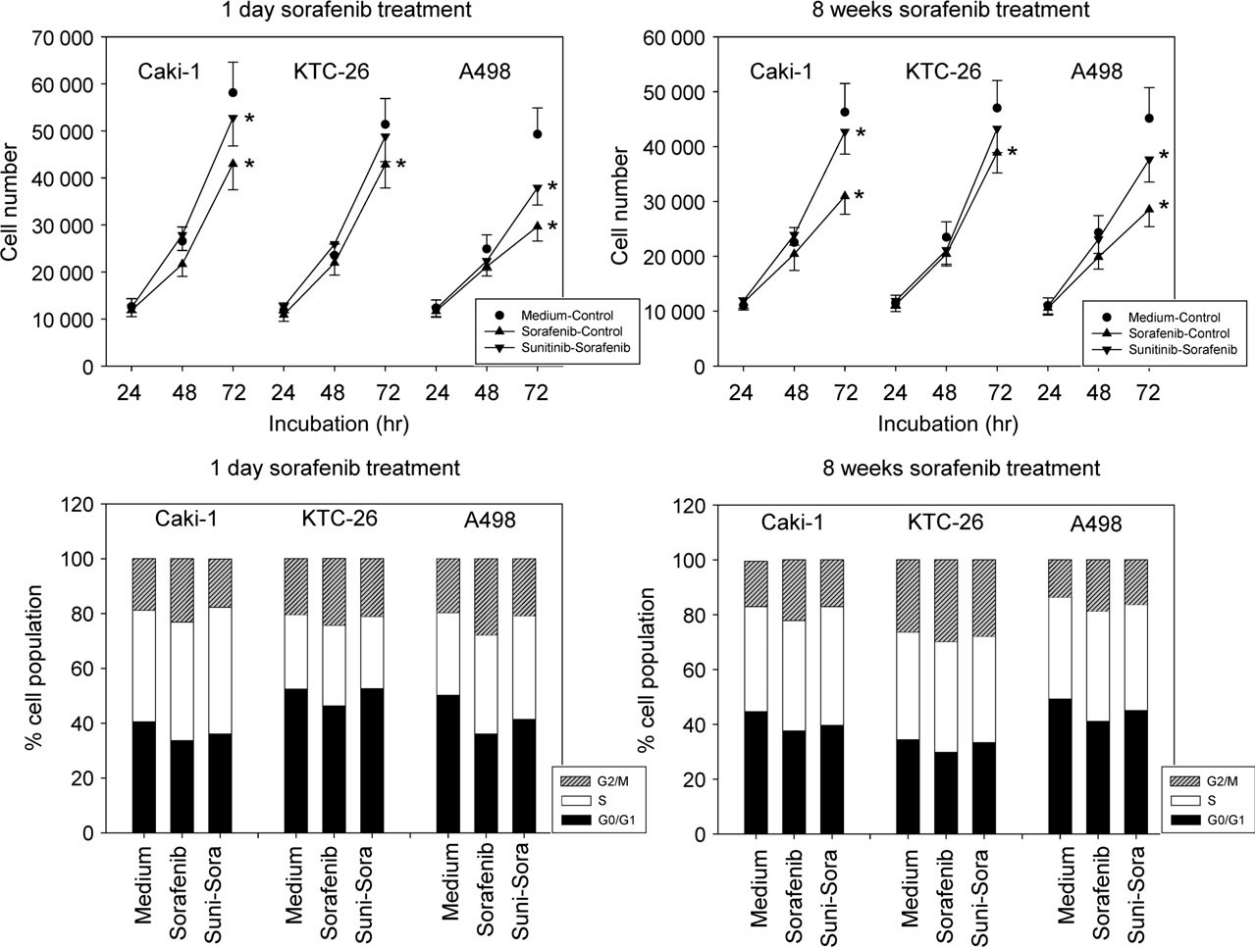
(Upper) Evaluation of tumour cell growth of sunitinib-resistant tumour cells exposed to sorafenib. Sunitinib resistant Caki-1, KTC-26 or A498 cells were treated short-term (1 day) or long-term (8 weeks) with 5 μM sorafenib (sunitinib-sorafenib). Controls were treated with culture medium alone (medium-control). To define the maximum effect of sorafenib, tumour cells not pre-treated with sunitinib were exposed to sorafenib (sorafenib-control). The figure shows one representative from six separate experiments. *indicates significant difference to controls. (Lower) Modulation of cell cycle progression in second line setting. Sunitinib resistant Caki-1, KTC-26 or A498 cells were treated short-term (1 day) or long-term (8 weeks) with 5 μM sorafenib (suni-sora). Controls were treated with culture medium alone (medium). To define the maximum effect of sorafenib, tumour cells not pre-treated with sunitinib were exposed to sorafenib (sorafenib). The cell population at each specific checkpoint is expressed as percentage of the total cells analysed. One representative experiment of three is shown.

Adding sorafenib to fresh (not pre-treated with sunitinib) tumour cells led to a significant reduction of tumour growth in Caki-1, KTC-26 and A498 cells. However, application of sorafenib to the sunitinib-resistant sublines did not cause growth-blockage in the KTC-26 model, whether the cells were treated with sorafenib for 1 day or chronically over 8 weeks (Fig.4, upper). Growth of sunitinib-resistant Caki-1 was only marginally influenced, and the down-modulating effect on the sunitinib-resistant A498 cells was not of the magnitude seen with the A498 cell controls.

Cell cycle analysis revealed that sorafenib, given to the controls (not pre-treated with sunitinib), significantly enhanced the number of Caki-1, KTC-26 and A498 cells in the G2/M-phase and significantly reduced the number of G0/G1-phase cells. In contrast, only a moderate action on cell cycle progression was induced when the sunitinib-resistant Caki-1 or A498 cells were treated with sorafenib. No influence was exerted by sorafenib in the sunitinib-resistant KTC-26 cell line, whether these cells were exposed to the compound for 1 day or 8 weeks (Fig.4, lower).

### Sequential switch to RAD001 following sunitinib-resistance

**Fig 5:**
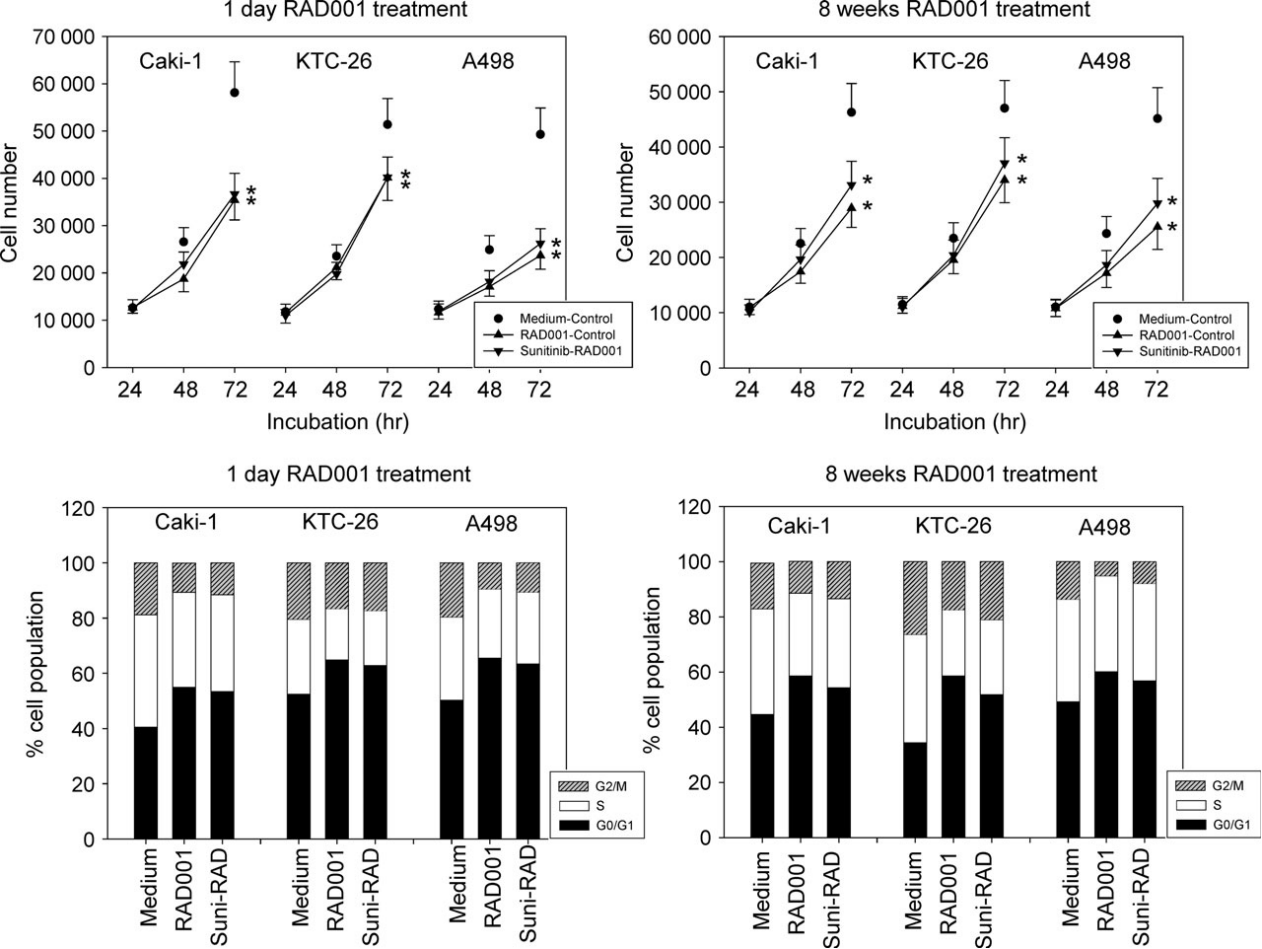
(Upper) Evaluation of tumour cell growth of sunitinib-resistant tumour cells exposed to RAD001. Sunitinib resistant Caki-1, KTC-26 or A498 cells were treated short-term (1 day) or long-term (8 weeks) with 5 nM RAD001 (sunitinib-RAD001). Controls were treated with culture medium alone (medium-control). To define the maximum effect of RAD001, tumour cells not pre-treated with sunitinib were exposed to RAD001 (RAD001-control). The figure shows one representative from six separate experiments. *indicates significant difference to controls. (Lower) Modulation of cell cycle progression in second line setting. Sunitinib resistant Caki-1, KTC-26 or A498 cells were treated short-term (1 day) or long-term (8 weeks) with 5 nM RAD001 (suni-RAD). Controls were treated with culture medium alone (medium). To define the maximum effect of RAD001, tumour cells not pre-treated with sunitinib were exposed to RAD001 (RAD001). The cell population at each specific checkpoint is expressed as percentage of the total cells analysed. One representative experiment of three is shown.

RAD001 strongly inhibited tumour growth in the control tests (cell lines not pre-treated with sunitinib) and exerted the same growth blocking potential on sunitinib-resistant cells, when applied for 1 day (Fig.5, upper left). RAD001 also considerably diminished the tumour cell number following a chronic 8 week exposure. However, slight differences to the controls (cell lines not pre-treated with sunitinib) were then seen (Fig.5, upper right).

RAD001 induced a significant G0/G1-phase increase, whereas the number of G2/M- (Caki-1, A498) and S-phase cells (KTC-26) was significantly decreased by RAD001 in control experiments (tumour cells not pre-treated with sunitinib). Short-term use of RAD001 (1 day) evoked similar effects on the sunitinib-resistant RCC cells (Fig.5, lower left). A significant cell cycle influence was obvious even after 8 weeks RAD001 exposure though there was then a slight loss of the drug's activity, compared to control tests (Fig.5, lower right).

### Cell cycle protein expression in the presence of sorafenib

**Fig 6:**
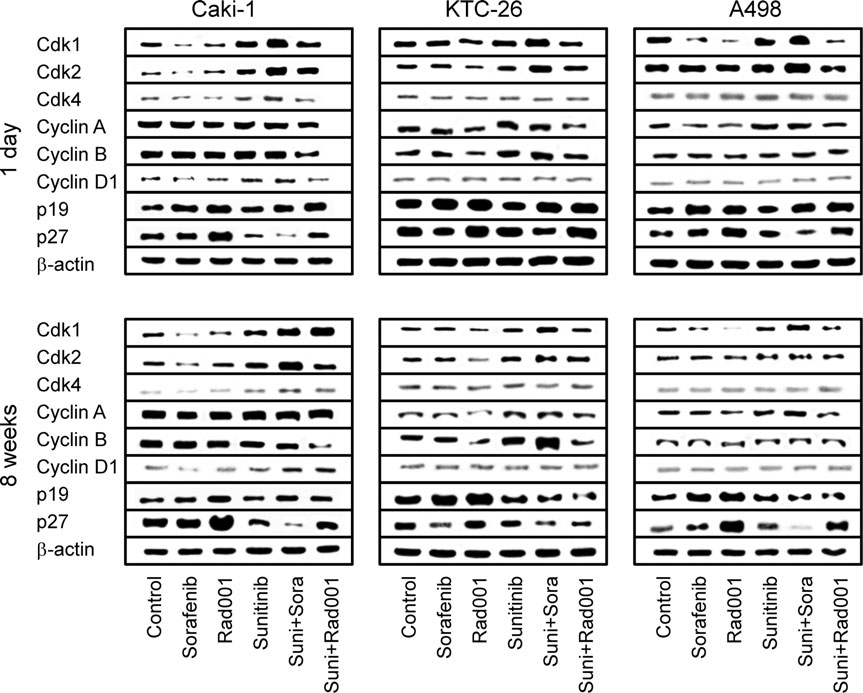
Analysis of cell cycle regulating proteins in Caki-1, KTC-26 and A498 cells after switching to sorafenib *versus*RAD001 treatment. Sunitinib resistant RCC cells were treated short-term (1 day) or long-term (8 weeks) with 5 μM sorafenib (suni+sora) or 5 nM RAD001 (suni+RAD001). Controls were treated with culture medium alone (control). To define the maximum effect of sorafenib and RAD001, tumour cells not pre-treated with sunitinib were exposed to sorafenib (5 μM; sorafenib) or RAD001 (5 nM; RAD001). Sunitinib resistant cells, which were further treated with sunitinib for 1 day or 8 weeks were also subjected to the assay (sunitinib). β-actin served as the internal control. The figure shows one representative from three separate experiments.

To define the maximum efficacy of sorafenib, sensitive tumour cells (not pre-treated with sunitinib) were exposed to sorafenib (‘sorafenib’) and protein expression was compared to that in untreated cells (‘control’). Evaluation of cell cycle regulating proteins in the control cells pointed to modulations of the cdk, p19 and p27 proteins in the presence of sorafenib. Caki-1 cells typically lost cdk1 and cdk2 while p19 was enhanced under sorafenib. There was also a loss of cdk1 in A498 cells, along with up-regulation of p19 and p27. Sorafenib caused an increase in the p19 level in KTC-26 cells, however, no changes were induced on cdk proteins in this cell line (Fig.6).

Subsequently, tumour cells were driven to sunitinib-resistance and then further treated with sorafenib for 1 day or for 8 weeks (‘suni+sora’). The protein content was compared with the protein expression of the sunitinib-resistant cells, which were further treated with sunitinib for 1 day or for 8 weeks (‘sunitinib’). The sunitinib-resistant tumour cell response to sorafenib was different from the control cell response, in as much as cdk1 and cdk2 were elevated and p27 was reduced over controls in Caki-1 cells. Cdk1 and cdk2 were also strongly elevated through sorafenib after 1 day in sunitinib-resistant KTC-26 cells (Fig.6, day 1). This effect was induced in KTC-26 cells after chronic use of sorafenib over 8 weeks as well, however, in an attenuated fashion. Instead, cyclin B was massively up-regulated, and p19 no longer increased the control protein level (Fig.6, 8 weeks). In sunitinib-resistant A498 cells, cdk1 and cdk2 were also enhanced after 1 day sorafenib application. Additionally, p27 was diminished, compared to the controls (Fig.6, day 1). The influence of sorafenib on cdk2 was lost after 8 weeks accompanied by cyclin A up-regulation and p19 reduction (Fig.6, 8 weeks).

To summarize, sorafenib added to sunitinib-sensitive tumour cells reduced cdk1 and cdk2 and enhanced p19 and p27 (with slight differences among the cell lines), whereas sorafenib added to sunitinib-resistant cells time-dependently elevated cdk1 and cdk2 and down-regulated p19 and p27.

### Cell cycle protein expression in the presence of RAD001

Treatment of the control cell lines (not pre-treated with sunitinib) with RAD001 resulted in an increase of the proteins p19 and p27 and a decrease of cdk1 in all control cell lines. There was also a reduction of cdk2, cyclin A and cyclin B in KTC-26 but not in Caki-1 and A498 cells (Fig.6).

Analogous to the sorafenib protocol, tumour cells were driven to sunitinib-resistance and then treated with RAD001 for 1 day or for 8 weeks (‘suni+RAD001’). The protein content was compared with the protein expression of the sunitinib-resistant cells which were treated with sunitinib for 1 day or for 8 weeks (‘sunitinib’). In doing so, the same alterations in p19 and p27 (all cell lines) or cyclin A and cyclin B (KTC-26 cells), as has been induced in the control experiments, were also induced when RAD001 was added for 1 day to the RCC cells pre-treated with sunitinib (Fig.6, 1 day). RAD001 additionally diminished cyclin A in sunitinib pre-treated A498 cells, and both cyclin A and cyclin B were diminished in sunitinib pre-treated Caki-1 cells. Extending the RAD001 application period to 8 weeks evoked further modifications. The expression level of cyclin A and p19 in the sunitinib pre-treated Caki-1 cells was similar to the controls, whereas cyclin B was reduced much more strongly by RAD001, compared to the 1 day application protocol (Fig.6, 8 weeks). In sunitinib pre-treated KTC-26 cells, RAD001 no longer elevated p19 and p27 (even a slight diminution occurred) but very strongly down-regulated cyclin B. In a similar manner, RAD001 slightly diminished p19 in the sunitinib pre-treated A498 cells under long-term conditions but led to a distinct loss of cyclin A (Fig.6, 8 weeks).

**Fig 7:**
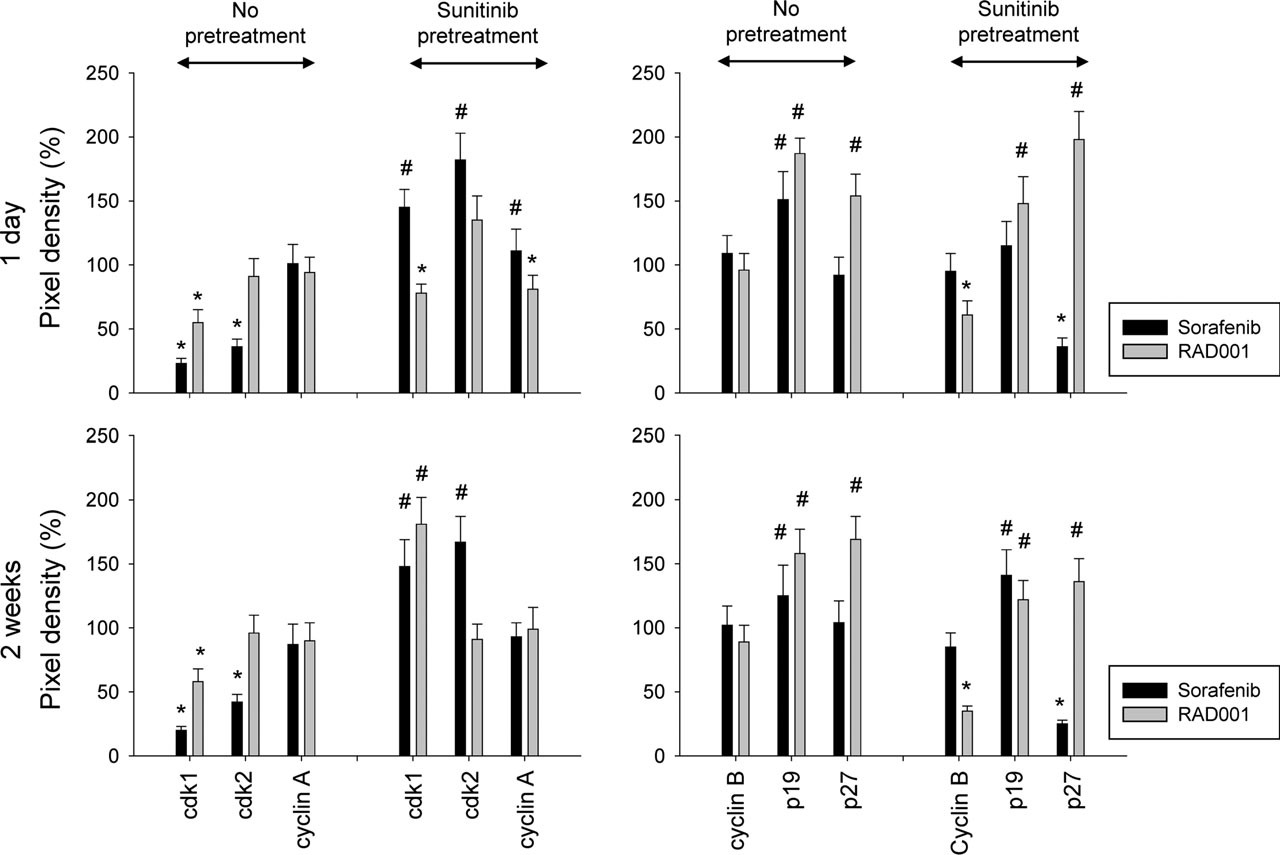
Quantification of cell cycle regulating protein expression in Caki-1 cells. Tumour cells not pre-treated with sunitinib were exposed to sorafenib or RAD001, or tumour cells were pre-treated with sunitinib for 8 weeks and then switched to sorafenib or RAD001. Pixel density is given in percentage related to controls not pre-treated with sunitinib (‘no pre-treatment’) or related to tumour cells pre-treated with sunitinib for 8 weeks (‘sunitinib pre-treatment’). *indicates significant decrease, #indicates significant increase related to the medium control in the ‘no pre-treatment’ assay or related to the sunitinib control in the ‘sunitinib pre-treatment’ assay.

To quantify protein expression in Caki-1 cells, pixel density analysis was performed. Figure7 demonstrates band intensities as percentage difference between control tumour cells (not pre-treated with sunitinib) and tumour cells treated with sorafenib or RAD001 (indicated as ‘no pre-treatment’) or as percentage difference between tumour cells pre-treated with sunitinib and tumour cells subsequently treated with sorafenib or RAD001 (indicated as ‘sunitinib pre-treatment’).

To summarize, RAD001 added to sunitinib-sensitive tumour cells reduced cdk1 and up-regulated p19 and p27. The same effects were induced when RAD001 was added to sunitinib-resistant cells. However, over time p19 began to diminish under the influence of RAD001.

### Intracellular signalling

**Fig 8:**
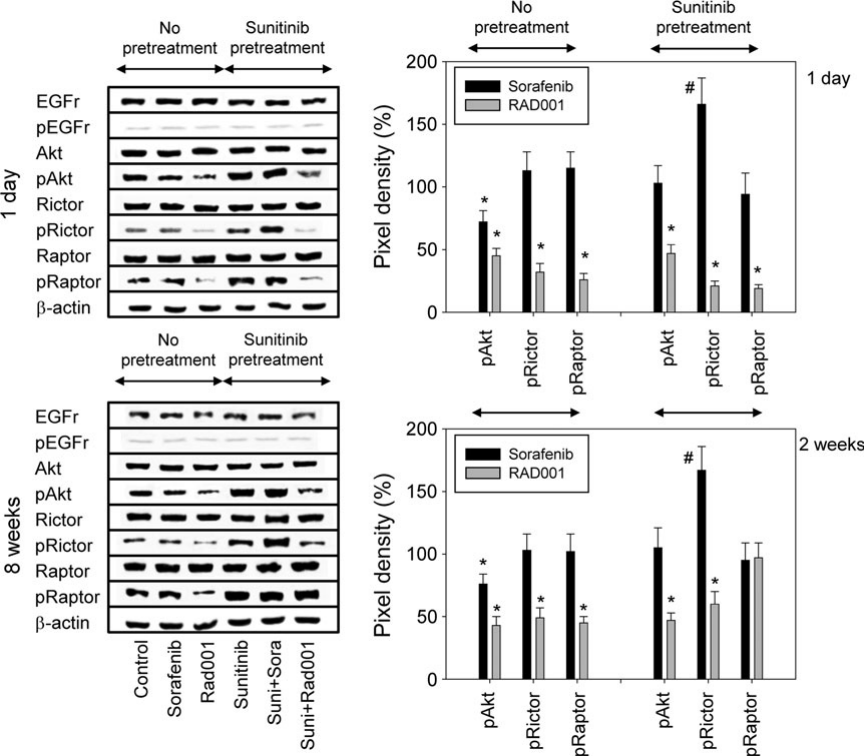
(Left) Modulation of cell signalling proteins following second line treatment. Sunitinib resistant Caki-1 cells were treated short-term (1 day) or long-term (8 weeks) with 5 μM sorafenib (‘suni+sora’) or 5 nM RAD001 (‘suni+RAD001’) and compared to sunitinib-resistant cells (8 weeks pre-treatment) which were further treated with sunitinib for 1 day or for 8 weeks (‘sunitinib’). To define the maximum effect of sorafenib and RAD001, tumour cells not pre-treated with sunitinib were exposed to sorafenib (‘sorafenib’) or RAD001 (‘RAD001’). Controls were treated with culture medium alone (‘control’). β-actin served as the internal control. The figure shows one representative from three separate experiments. (Right) Quantification of cell signalling protein expression in Caki-1 cells. Tumour cells not pre-treated with sunitinib were exposed to sorafenib or RAD001, or tumour cells were pre-treated with sunitinib for 8 weeks and then switched to sorafenib or RAD001. Pixel density is given in percentage related to controls not pre-treated with sunitinib (‘no pre-treatment’) or related to tumour cells pre-treated with sunitinib for 8 weeks (‘sunitinib pre-treatment’). *indicates significant decrease, #indicates significant increase related to the medium control in the ‘no pre-treatment’ assay or related to the sunitinib control in the ‘sunitinib pre-treatment’ assay.

Analysis of intracellular signalling, which was carried out on Caki-1 cells, did not reveal an influence of sorafenib on the control cells. However, the mTOR unit, pRictor, was enhanced when sunitinib was followed by sorafenib in both the 1 day and 8 weeks protocol (Fig.8).

The target proteins of RAD001, Akt, Rictor and Raptor, were considerably deactivated by this drug in the control experiments (Fig.8). RAD001 (1 day) was equally effective in down-regulating phosphorylated Akt, Rictor and Raptor in Caki-1 cells pre-treated with sunitinib (Fig.8, 1 day). Even after 8 weeks of RAD001 application, Caki-1 cells were characterized by a strong loss of pRictor and pAkt (Fig.8, 8 weeks).

The right panel of Figure8 depicts band intensities calculated for pAkt, pRictor and pRaptor. Intensity is shown as percentage difference between control tumour cells (not pre-treated with sunitinib) and tumour cells treated with sorafenib or RAD001 (indicated as ‘no pre-treatment’) or as percentage difference between tumour cells pre-treated with sunitinib and tumour cells subsequently treated with sorafenib or RAD001 (indicated as ‘sunitinib pre-treatment’).

### Apoptosis

The Annexin V-FITC Apoptosis Detection kit did not reveal distinct signs of apoptosis in tumour cells. Early and late apoptosis of Caki-1 cells were always below 5%. The same was true with respect to Bax and Bcl-2 expression. No caspase-3 cleavage was observed in cells treated under any conditions (data not shown). Additionally, cell viability, which was also controlled during the investigation, did not reveal enhanced cell death (data not shown).

## Discussion

Sunitinib is employed in first-line treatment of RCC 9 and therefore was also employed as the first-line application in this *in vitro* investigation. Short-term application of sunitinib diminished growth in all RCC cell lines. Since members of the cdk and cyclin family were reduced in parallel, modifications of the cdk-cyclin axis (particularly cdk1-cyclin B and cdk2-cyclin A) may be one mechanism by which sunitinib exerts its antitumour effect. No pertinent data concerning solid tumours is available but sunitinib has been demonstrated to diminish cdk2 in acute myeloid leukaemia cells 10. Sunitinib up-regulated p19 and p27 in RCC cells, whereby the role of p27 has been controversially discussed. Immunohistochemical analysis of renal cancer tissue has revealed a high cytoplasmic p27 level associated with advanced disease and reduced cancer specific survival 11. This was not seen in another immunohistochemical study, where a low p27 expression correlated with poor outcome 12. The difference between the investigations was that one was based on the tissue microarray technique 11, and the other 12 was based on microscopic evaluation of the p27 staining intensity. Western blot evaluation of A498 cells has demonstrated an inverse relationship between cell growth and p27 expression *in vitro* 13,14. Therefore, p27 elevation may reflect a specific mechanism of sunitinib that slows down the cell cycle. The same might hold true for p19, and since both p27 and p19 serve as cdk-inhibitors, it seems likely that up-regulation of p19 and p27 is directly coupled to the inhibition of cdk1 and cdk2 15.

Recently, cross-communication between Akt and the p27-cdk axis has been reported 16,17. In this context, sunitinib suppressed pAkt in Caki-1 cells, a phenomenon which has also been observed in the RCC cell line ACHN following sunitinib exposure 18. However, Akt alterations were only minor and the downstream targets Rictor and Raptor were not influenced by sunitinib. Sunitinib's effect, therefore, differs from the early effects of RAD001, which have been characterized by a strong reduction of pAkt, pRictor and pRaptor. Whether Akt deactivation, caused by sunitinib, is a clinically relevant mechanism or just a minor side-effect is not yet clear.

Long-term sunitinib administration led to the development of distinct feedback loops. As a principal mechanism (with slight differences among the cell lines), cdk1/cdk2 and the respective binding partners cyclin B/A became enhanced, compared to the controls, and p19 and p27 were no longer elevated, as seen under short-term treatment.

Recently, the cdk1/2-cyclin A/B complex has been identified as critically involved in resistance development of prostate cancer cells 19. There is also evidence that forced expression of cdk1-cyclin B attenuates drug sensitivity in glioma cells 20, and cyclin B overexpression in patients with head and neck squamous cell carcinoma has been correlated with poor therapeutic response 21. We assume that up-regulation of cdk1 and cdk2 together with their counterparts cyclin A and B may reflect a specific resistance mechanism seen during sunitinib therapy. The expression level of cdk-cyclin could, therefore, serve as a potential clinical marker to predict sunitinib sensitivity. The same might be true for Akt and its down-stream target Rictor, which are both reactivated in Caki-1 cells under long-term sunitinib exposure. In good corroboration, sunitinib resistance in the RCC cell line 786-O has been shown to be accompanied by up-regulation of pAkt 22. No sunitinib induced modification of Raptor, which is the main target of RAD001, was found.

When interpreting the antitumour mechanism of sunitinib, we should be aware that VEGFR was not detected in Caki-1 cells. Presumably, sunitinib interacts with another receptor type. A positive correlation between the sensitivity to sunitinib and PDGFR-β has recently been demonstrated 23. Menke *et al*. identified CSF-1R as a major regulator of RCC survival and proliferation, and blocking this receptor by a TKI dramatically reduced *in vivo* tumour mass 24.

Once resistance was acquired to sunitinib, sorafenib or RAD001 was applied. The data presented here demonstrate that the RCC cells regained full responsiveness to the mTOR-inhibitor RAD001, whereas the TKI-inhibitor sorafenib was only partially effective in blocking tumour growth. Data from prospective studies are not yet available. However, Larkin and coworkers have shown that the sequence, sunitinib-RAD001, is superior to the sequence, sunitinib-sorafenib, in an orthotopic mouse model of RCC 25. Sorafenib exhibited a similar mode of action to that of sunitinib, whereby RCC cells, not pre-treated with sunitinib accumulated in the G2/M-phase of the cell cycle. This effect has also seen by others who have demonstrated an increased amount of Caki-1 and 786-O cells in G2/M following sorafenib application 26. Molecular analysis points to similarities in regulating cdk1, cdk2, p19 and p27, at least in Caki-1 and A498 cells. Finally, pAkt was diminished in Caki-1 by sorafenib, but not Rictor and Raptor. Since the same regulatory mechanism is evoked by sunitinib and sorafenib, the limited efficacy of the sunitinib-sorafenib regimen might be due to cross-resistance. In fact, up-regulated cdk1 and cdk2 and down-regulated p27 under long-term sunitinib treatment were further elevated (cdk1, cdk2) or further diminished (p27), when sunitinib was replaced by sorafenib. In line with this, RCC cells with acquired refractoriness to sunitinib have been demonstrated to develop cross-resistance to sorafenib 18.

These results are derived from a cell culture model, which may not adequately reflect the clinical situation. However, retrospective analysis of RCC patients treated with sequential sunitinib-sorafenib or sorafenib-sunitinib has shown a worse outcome in the sunitinib-sorafenib group, indicating that at least a limited cross-resistance might exist in the TKI-TKI schedule 27,28. Data from prospective trials are now required to finally assess the risk of cross-resistance in the clinical setting.

The mode of action of RAD001 is different from that of sunitinib. RAD001 leads to a cell growth delay in G0/G1 and diminished pAkt along with pRaptor and pRictor. mTOR is found in two different complexes within the cell, mTORC1 (containing Raptor) and mTORC2 (containing Rictor), but only mTORC1 is thought to be sensitive to inhibition by RAD001 29. The theory of selective Raptor targeting might, therefore, be challenged. In fact, RAD001 has recently been reported to interfere with the assembly of both mTOR complexes, mTORC1 and mTORC2, in acute myeloid leukaemic cells 30,31.

Presumably, the different molecular activity of sunitinib and RAD001 may be the reason why RAD001 evoked full response in the sunitinib-resistant RCC cells. Molecular evaluation has furnished evidence that high p27 expression, which was lost under sunitinib-resistance (and further diminished under second line sorafenib) was restored in the presence of RAD001. RAD001 also counteracted the process of cdk1 and cdk2 up-regulation induced by chronic sunitinib administration. Most impressively, mTOR signalling was blocked by RAD001 in the sunitinib-resistant cancer cells with the same strong potency as was seen in the control assays. mTOR represents the master regulator of cell proliferation. Therefore, it is not astonishing that RAD001 stopped tumour growth of sunitinib-resistant and control cancer cells equally well. Logically, the introduction of an mTOR-inhibitor for second-line therapy could be the strategy of choice after failure of first-line sunitinib therapy. This is supported by an *in vivo* investigation demonstrating significant antitumour and anti-metastatic effects in mice transplanted with renal cancer cells under sequential therapy with sunitinib, followed by RAD001 25. In good accordance, Rosa *et al*. demonstrated that as single agents, sunitinib, sorafenib and RAD001 share similar activity in inhibiting cell proliferation in different RCC models, whereas pre-treatment with sunitinib reduced the response to subsequent sorafenib application but not to RAD001 application 32.

Recently, a retrospective study was conducted to evaluate treatment outcome associated with common second-line targeted therapies, given after first-line sunitinib for metastatic RCC. The adjusted risk of treatment failure differed between second-line molecular-targeted therapies, being 1.8 times higher for sorafenib than for RAD001 33. Another retrospective study, selected from the RECORD-1 trial, has suggested that sunitinib-refractory metastatic RCC patients treated with everolimus may experience significantly improved overall survival, compared to those treated with sorafenib 34.

Enhanced apoptosis was not detected in tumour cells treated with sunitinib or sorafenib. This may not be true *in vivo*, but since we did not carry out *in vivo* experiments, it is impossible at this point to speculate on that situation. However, other *in vitro* investigations also do not detect enhanced apoptosis. Shablak *et al*. and Kususda *et al*. recently reported that sunitinib and sorafenib do not trigger apoptosis in RCC cell lines *in vitro* 35,36. Likewise, RAD001 has also been shown not to induce apoptosis in RCC cells *in vitro* 7.

In conclusion, RAD001 was shown to be superior to sorafenib in second-line application. However, long-term exposure of the sunitinib-refractory tumour cells with RAD001 slightly reversed expression and activity of some target proteins. The up-regulation of p27 in Caki-1 and A498 cells was not as strong as that after starting the RAD001 therapy. Notably, Raptor was no longer deactivated by RAD001 after 8 weeks, suggesting that early signs of resistance may have developed. This may explain why long-term exposure of the sunitinib-refractory tumour cells with RAD001 exhibited slightly diminished growth-blocking potential, compared to short-term growth-blocking potential. It seems likely that long-term use of RAD001 induces a counter-regulatory mechanism, leading to drug non-responsiveness. Perhaps, switching back to a TKI with a different molecular mode of action could then be employed to overcome RAD001 refractoriness. However, this is speculative and warrants further investigation.
